# A Table of the Greatest, Least, and Mean Heights of the Thermometer and Barometer, and of the Fall of Rain in London in 1779

**Published:** 1781-02

**Authors:** 


III. *A TABLE of the Greatest, Least, and Mean Heights of the Thermometer and Barometer, and of the Fall of Rain in London in* 1779. (*From the* Philosophical Transactions, Vol. 70, Part I.)

| 1779. | Thermometer without. | | | Thermometer within. | | | Barometer. | | | Rain. |
|---|---|---|---|---|---|---|---|---|---|---|
| | Greatest Height. | Least Height. | Mean Height. | Greatest Height. | Least Height. | Mean Height. | Greatest Height. | Least Height. | Mean Height. | Inches. |
| January | 50,0 | 25,0 | 37,2 | 48,5 | 31,5 | 38,2 | 30,61 | 29,63 | 30,24 | 0, 216 |
| February | 60,5 | 37,0 | 47,9 | 53,5 | 44,0 | 48,8 | 30,53 | 29,84 | 30,26 | 0, 239 |
| March | 73,0 | 33,0 | 49,2 | 56,0 | 35,0 | 48,1 | 30,63 | 29,63 | 30;19 | 0, 523 |
| April | 78,0 | 38,0 | 53,8 | 64,0 | 47,0 | 54,8 | 30,59 | 29,43 | 29,26 | 1, 224 |
| May | 82,5 | 41,5 | 58,4 | 79,0 | 45,0 | 59,4 | 30,27 | 29,51 | 29,82 | 1, 708 |
| June | 75,0 | 52,5 | 61,7 | 69,0 | 51,5 | 64,0 | 30,22 | 29,48 | 29,92 | 2, 476 |
| July | 85,0 | 57,0 | 68,0 | 81,0 | 63,0 | 69,5 | 30.42 | 29,30 | 29,91 | 6, 401 |
| August | 84,5 | 55,0 | 67,4 | 79,0 | 62,0 | 69,4 | 30,29 | 29,64 | 29,05 | 1, 074 |
| September | 79,0 | 52,0 | 63,4 | 78,0 | 58,0 | 65,3 | 30,20 | 29,54 | 29,87 | 2, 232 |
| October | 64,5 | 36,0 | 54,2 | 88,0 | 43,5 | 56,8 | 30,42 | 29,54 | 29,96 | 3, 031 |
| November | 69,5 | 29,0 | 44,2 | 85,5 | 35,5 | 45,0 | 30,39 | 28,75 | 29,62 | 2, 778 |
| December | 58,0 | 20,0 | 42,2 | 58,5 | 28,0 | 42,2 | 30,33 | 28,85 | 29,50 | 4, 883 |
| Whole Year | | | 53,9 | | | 155, | | | 29,43 | 26,785 |

